# Advances in the Management of Craniopharyngioma: A Narrative Review of Recent Developments and Clinical Strategies

**DOI:** 10.3390/jcm14041101

**Published:** 2025-02-09

**Authors:** Mousa Javidialsaadi, Diego D. Luy, Heather L. Smith, Arba Cecia, Seunghyuk Daniel Yang, Anand V. Germanwala

**Affiliations:** 1 Department of Neurological Surgery, Loyola University Medical Center, Maywood, IL 60153, USA; 2 Department of Pathology, Loyola University Medical Center, Maywood, IL 60153, USA

**Keywords:** craniopharyngioma, papillary, adamantinomatous, review, Rathke’s pouch, parasellar

## Abstract

Craniopharyngiomas (CPs) are rare intracranial tumors arising from remnants of Rathke’s pouch. Despite their benign histology, CPs present considerable clinical challenges due to their tendency to exert mass effect and adherence to critical neurovascular structures. There remains no clear consensus on the most effective management of CPs. We explore the latest developments in targeted treatment approaches, examining how innovations in surgery, radiation therapy, and emerging therapies are improving outcomes and management for these challenging tumors. In addition to providing our experience, we reviewed previously reported case series and reviews relevant to CPs. Studies show a bimodal age distribution for CP diagnosis, with peak incidence occurring in children aged 5–14 years and in adults aged 50–74 years. Surgical resection is the typical initial treatment for CPs, and adjuncts, including radiation therapy and intracavitary treatments, have been proven effective for tumor control. Additionally, genetic mutations associated with CPs offer an opportunity for novel strategies that address the underlying molecular mechanisms driving tumor growth through targeting the Wnt/β-catenin and/or MAPK/ERK pathways to disrupt the aberrant signaling that promotes tumor proliferation and survival. Survival rates for CPs are generally favorable (five-year survival of 80%), with recent studies showing improved outcomes and higher survival rates in children. CPs remain rare and challenging tumors. Although surgical resection is the main treatment, surgeons must weigh the benefits of achieving a gross total resection with the risks of iatrogenic injury. Adjuncts, including intracavitary and radiation treatments, may assist with subtotal resections and recurrences, as well as approved BRAF inhibitor therapy for the papillary variant. Many improvements in diagnostic and therapeutic methods were made after Dr. Cushing coined the term “craniopharyngioma”. Ongoing experiments, investigations, and advances in radiation techniques and molecular targeted therapies will provide patients with promise for safer and more effective treatments.

## 1. Introduction

Almost a century has elapsed since the initial surgical attempt to remove a craniopharyngioma (CP), yet managing patients with these tumors remains a significant challenge and continues to test the expertise of those involved in their treatment [1]. CPs are rare intracranial tumors arising from remnants of Rathke’s pouch, typically situated in the sellar/parasellar region [2,3]. Despite their benign histology, CPs present considerable clinical challenges due to their tendency to compress and adhere to nearby neurovascular structures, particularly impacting the hypothalamic–pituitary axis, and create significant endocrinologic disturbances such as growth hormone deficiency and diabetes insipidus [4,5]. Epidemiological studies show a bimodal age distribution with peak incidence occurring in children aged 5–14 years and in adults aged 50–74 years [6]. They account for 1.2–4.6% of all brain tumors, with an incidence rate of 0.5–2.5 new cases per 1 million people [7]. Although the 5-year survival rate for CPs is 89–94% and the 10-year survival rate is 85–90%, their high recurrence rate frequently necessitates a combination of treatments to effectively manage the tumor [8]. These tumors are categorized into two primary subtypes, adamantinomatous CP (ACP) and papillary CP (PCP), each characterized by distinct genetic profiles, clinical behaviors, and prognostic outcomes [9,10]. ACPs are typically cystic and solid while PCPs are usually solid and lack calcifications on imaging. Current treatment approaches predominantly involve surgical resection and radiosurgery aimed at achieving tumor control; however, these methods carry inherent risks, including high recurrence rates and potential morbidity [11,12,13]. For instance, due to their notorious adherence to neurovascular structures, purely surgical approaches often result in unacceptable morbidity [14]. Addressing these challenges necessitates ongoing research into innovative therapeutic strategies to enhance treatment efficacy and minimize adverse effects, highlighting continual evolution in the management of CPs. The fundamentals of CP management emphasize achieving an optimal balance between high local control and limited long-term toxicity [15].

As a primary objective of this review, we highlight the current understanding of craniopharyngiomas, including their epidemiology, mutational profile, clinical presentation, varying histopathology, and treatments. As a secondary objective, we explore the latest developments in targeted treatment approaches and offer our multidisciplinary strategy in the management for these challenging tumors.

## 2. History

In 1857, Friedrich Albert Von Zenker made a pioneering description of CPs, identifying clusters of cells resembling squamous epithelium located in the pars tuberalis and pars distalis of the pituitary gland [16]. Jacob Erdheim significantly advanced our understanding in 1904 by characterizing these lesions as hypophysial duct tumors (Hypophysenganggeschwülste), a term to describe their origin from cellular remnants of the hypophyseal duct, through which the Rathke’s pouch migrates to help form the pituitary gland. Erdheim’s detailed histopathological studies revealed that CPs in adults frequently appear on the anterior surface of the infundibulum, manifesting as groups or islets of various sizes and shapes [17]. Subsequent research in 1932 by Susman identified squamous epithelial cell nests within the pituitary glands of children, marking a critical discovery in the tumor’s pathology [18]. A milestone in treatment was achieved in 1909 when A. E. Halstead performed the first successful surgical resection of a CP, paving the way for modern surgical approaches [19]. Harvey Cushing, a pivotal figure in neurosurgery, advocated for the transcranial approach specifically for CP surgeries, a departure from his preferred transsphenoidal approach for other pituitary surgeries [20]. Cushing introduced the term Craniopharyngioma in 1932 to encompass a heterogeneous group of tumors arising from the Rathke’s pouch [21]; the term not only standardized its nomenclature throughout the literature but also contributed significantly to advancing the understanding and management of this complex group of Rathke’s pouch tumors [22].

## 3. Epidemiology of Craniopharyngiomas

### 3.1. Age Distribution and Bimodal Peak

CPs exhibit a distinctive bimodal age distribution, characterized by two primary peaks in incidence. The first peak occurs in children aged 5 to 14 years, reflecting a significant incidence rate during early childhood and adolescence. The second peak is observed in adults aged 50 to 74 years, indicating a resurgence of cases later in life [3,23,24]. An analysis of data from the United States spanning from 2004 to 2016 corroborates this pattern, revealing peak incidence rates at ages 5 to 9 years and 65 to 69 years [25]. This bimodal distribution is not only consistent across multiple studies but also underscores the substantial impact of age on the occurrence of CPs [26,27]. The persistence of these peaks suggests underlying biological or environmental factors that contribute to the disease’s age-related incidence. ACPs are typically found in both peak incidences (children and adults), while PCPs are classically found in the second peak (adults only) [7].

The prevalence of multicystic tumors is notably higher in childhood-onset cases compared to adult-onset cases, a trend confirmed by Wijnen et al. (2017), who reported a greater frequency of multicystic tumors in childhood-onset cases (45% vs. 21%, *p* = 0.02) [28]. Additionally, hydrocephalus and third ventricle involvement are more frequently observed in childhood-onset cases, with 62.5% of these patients exhibiting hydrocephalus compared to 35.5% of adult-onset patients, a finding that is consistent with observations from other studies [28,29].

### 3.2. Global Incidence and Prevalence

CPs represent approximately 10% of primary brain tumors in the pediatric population, underscoring their significance in the field of neuro-oncology [30]. Global incidence rates for CPs vary widely, with reported figures ranging from 0.09 to 0.57 per 100,000 person-years [24]. In the United States, data from the Central Brain Tumor Registry of the United States (CBTRUS) and Los Angeles County indicate an overall incidence rate of 0.13 per 100,000 person-years, translating to roughly 338 new cases annually, including 96 cases in children aged 0 to 14 years [25]. From 2004 to 2016, a total of 6643 cases were diagnosed, corresponding to an average of about 600 new cases each year and an incidence rate of 0.16 per 100,000 persons [25]. More recent estimates have suggested a prevalence rate of 5.27 per 100,000 people [31], further enriching the data on the prevalence of CPs and highlighting their ongoing impact on the population [32].

### 3.3. Subtypes and Their Prevalence

Among the various subtypes of CPs, ACPs are the most prevalent, accounting for approximately 81% of all cases [33]. In contrast, PCPs constitute a smaller proportion of cases, with their prevalence varying significantly across different age groups. Specifically, PCPs are found in 5.5% of individuals aged 0–29 years, 30.6% of those aged 30–59 years, and 30.4% of individuals aged 60 years and older [25]. This age-related variation underscores the relative rarity of PCPs compared to the more common ACPs subtype.

### 3.4. Demographic Disparities

Incidence rates of CPs reveal notable demographic differences. In the United States, the highest incidence is observed among Black individuals, with a rate of 0.22 per 100,000, followed by White individuals at 0.15 per 100,000, Asian/Pacific Islander populations at 0.14 per 100,000, and American Indian/Alaska Native populations at 0.10 per 100,000 [25]. Gender and Hispanic ethnicity do not show significant variations in incidence rates [25]. These demographic disparities underscore the need for targeted research to explore and understand the underlying factors contributing to these variations in CP incidence.

### 3.5. Diagnostic and Treatment Trends

CPs are initially treated with surgical intervention, with advancements in imaging and surgical techniques significantly enhancing the rates of GTR, which vary globally from 17% to 89% [30]. For cases where GTR is not feasible, subtotal resection (STR) combined with adjuvant radiation therapy is commonly employed to address recurrence and minimize morbidity. Fractionated radiotherapy has shown high control rates, ranging from 92.0% to 100.0% in short-term follow-ups, particularly when used alongside modern imaging technologies such as CT and MRI [30].

### 3.6. Clinical Outcomes and Quality of Life

Overall survival rates for CP patients are generally favorable. In the United States, the 5-year survival rate is approximately 80%, with recent years showing improved outcomes and higher survival rates observed in children [25]. Long-term survival rates for CP patients vary significantly, with reports ranging from 40% to 95% at 10 years, according to recent studies [31,34,35,36,37,38]. The transsphenoidal approach is the most frequently employed surgical technique in contemporary practice [31]. This approach, through the nostrils, gumline, or nasal septum, aims to achieve effective tumor resection while preserving hypothalamic function and neurovascular structures [30].

## 4. Genetic Mutations in Craniopharyngiomas

CPs, though benign, are associated with distinct genetic alterations that play a crucial role in their development. In ACPs, the most frequent mutation involves the *CTNNB1* gene (3p21) [39,40], which encodes the protein β-catenin. Mutations in this gene lead to aberrant activation of the Wnt/β-catenin signaling pathway [41,42,43,44,45]. Activation of the Wnt- pathway has been shown, in some studies, to cause the upregulation of EGFR- and SHH- pathways and influence tumor cell migration. This dysregulation is fundamental to the pathogenesis of ACPs, influencing cellular proliferation and tumor growth [42]. However, targeting the Wnt/β-catenin signaling pathway remains a challenge, with very few existing molecular studies. One case report demonstrates some efficacy of Bevacizumab (vascular endothelial growth factor inhibitor) in reducing both the cystic and solid components of ACP [46]. Additionally, there is some evidence that the cystic and solid portions of ACPs contain interleukin 6 receptors, and treatment with Tocilizumab (interleukin 6 receptor antagonist) has been shown to decrease cyst burden [43,44,45].

Conversely, approximately 90% of PCPs are primarily characterized by mutations in the BRAF gene (7q34) [47], specifically, the BRAF V600E mutation [39,48]. This mutation results in the activation of the MAPK/ERK signaling pathway, which promotes tumor cell proliferation and survival [49,50]. BRAF V600E-MEK inhibitor combination therapy has shown promising results in PCP management. A phase 2, single-group study examined the combinatory effects of Vemurafenib (BRAF V600E inhibitor) and Cobimetinib (MEK inhibitor) on patients diagnosed with PCP. The study found that patients who received Vemurafenib–Cobimetinib combination therapy had a 91% median reduction in tumor volume. Progression-free survival at 12 and 24 months was determined to be 87% and 58%, respectively, with a 12-month overall survival of 100%. In addition, 93% of patients experienced continued tumor reduction at 12-month follow-up [48]. Another study investigating the combined effect of BRAF inhibitor (Debrafenib) and MEK inhibitor (Trametinib) found 75% tumor reduction by 5 months [51]. A summary of the key differences between the types of craniopharyngiomas is shown in Table 1.

The identification of these genetic mutations not only enhances our understanding of the distinct molecular mechanisms driving each CP subtype but also provides a basis for targeted therapeutic approaches. Advances in molecular diagnostics and targeted therapies could significantly impact the management of CPs, offering more individualized treatment options based on the specific genetic alterations present in these tumors.

## 5. Clinical Signs and Symptoms

CPs exhibit slow growth rates and, thus, symptoms associated with craniopharyngiomas have insidious onsets [52,53]. When symptomatic patients with CPs may experience headaches, visual deficits, nausea, vomiting, and endocrine disruptions given their associated mass effect upon the hypothalamic-pituitary gland axis (HPA), insult to the pituitary gland may result in disturbances of several hormones, including growth hormone (GH), gonadotropins (LH/FSH), adrenocorticotropic hormone (ACTH), vasopressin, and thyroid-stimulating hormone (TSH) [10,52,54]. These impairments may also be age-dependent, i.e., growth impairment in children and sexual dysfunction in adults [10,52,54]. Additional superior involvement of the infundibulum or chiasmatic extension may result in diabetes insipidus (DI) and bitemporal hemianopsia, respectively [52,54]. A retrospective review conducted at Aga Khan University Hospital in Karachi, Pakistan, from 2001 to 2020, identified common initial symptoms of CPs, including headache in 83.6% of cases and visual deficits in 81.6% [33].

More rare presentations with seizures, mood dysregulations, weakness, hallucinations, and autonomic disturbances have also been reported [10,52]. A lateral extension of CPs may also exert mass effect upon cavernous sinus contents, including cranial nerves (CN) III (oculomotor nerve), CN IV (trochlear nerve), CN V1 (ophthalmic division of the trigeminal nerve), CN V2 (maxillary division of the trigeminal nerve), and CN VI (abducens nerve), resulting in limited extraocular muscle movement with resultant strabismus and diplopia [52]. The obstructive mass effect from large CPs may also result in hydrocephalus, papilledema, and optic disc atrophy with associated vision loss [55]. Table 2 summarizes the most common signs and symptoms.

## 6. Diagnosis and Management of Craniopharyngioma

CPs can occur at a variety of ages, including during the prenatal and neonatal periods [57,58]. Despite being classified as a benign tumor by the World Health Organization (WHO), CPs can lead to substantial morbidity and mortality due to their locally aggressive growth pattern [59]. Therefore, early and precise diagnosis is essential for creating effective treatment plans [60]. The management of congenital craniopharyngiomas (cCPs) presents unique challenges and underscores the need for prompt and effective intervention. As advances in prenatal imaging and surgical techniques continue to evolve, it is crucial to understand their impact on patient outcomes. A recent study sheds light on these aspects by examining the management of CPs in a cohort of 361 patients diagnosed with ACP between 2007 and 2024 [61]. The study highlighted the efficacy of early detection through prenatal ultrasound, facilitating timely surgical intervention within days of birth. Despite varying postoperative outcomes, including mild neurological deficits and functional impairments in some cases, all patients survived. These findings underscore the critical role of early diagnosis and multidisciplinary care in optimizing outcomes for these rare tumors.

Recent research and a focused study highlight the critical role of routine monitoring in the early detection of childhood-onset CP [62]. By tracking growth parameters such as weight development and head circumference, healthcare providers can gain valuable insights into potential abnormalities indicative of CP. This proactive approach enables earlier diagnosis, allowing for timely intervention before the tumor leads to more severe hypothalamic and pituitary stalk involvement. Early identification not only facilitates more targeted and effective treatment strategies but also significantly improves patient outcomes by reducing the risk of extensive complications and enhancing the overall quality of life [62].

**Figure 1:**
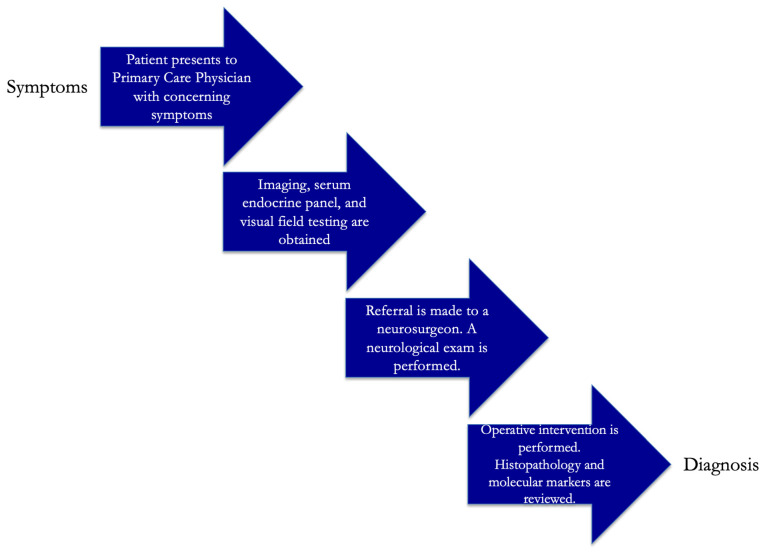
Typical multidisciplinary diagnostic algorithm is demonstrated.

Children and adults with concerning symptoms and signs typically present to primary care physicians. CT and MR imaging can typically reveal a sellar and suprasellar mass. While the CT can be used to evaluate for calcifications, the MRI delineates the cystic and solid components and the extent of the craniopharyngioma in more detail as well as the relationship of the tumor to the chiasm, pituitary stalk and gland, and hypothalamus. A serum endocrine panel is typically ordered to evaluate for hormonal deficiency; endocrinologists are consulted for any hormonal dysfunction noted on bloodwork. Ophthalmology referral for visual field testing is obtained to evaluate the function of the optic apparatus. An eventual referral is made to a neurosurgeon; the diagnosis is a tissue-proven one and mandates biopsy and/or resection (Figure 1).

## 7. Histopathologic Features and Radiologic Features

**Figure 2:**
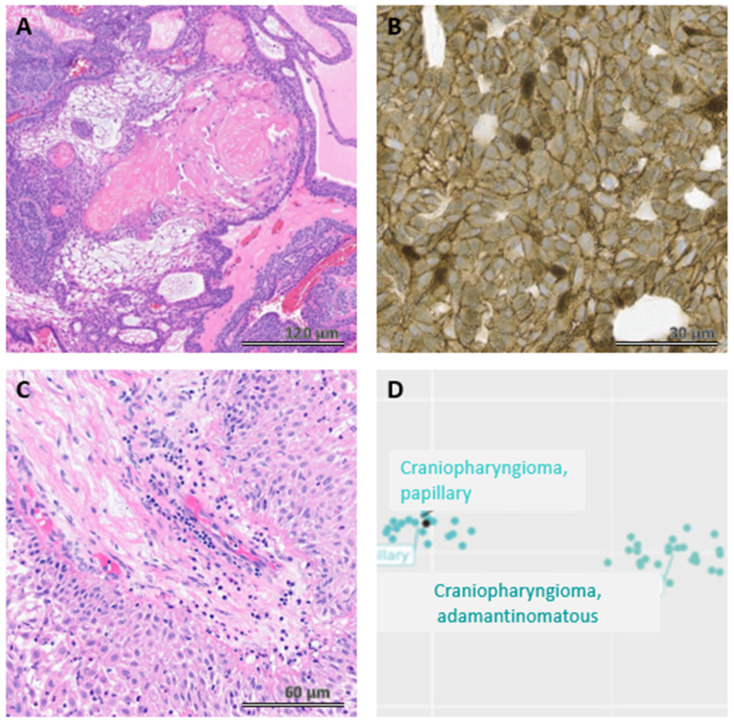
Adamantinomatous craniopharyngiomas (**A**) are composed of cords, whorls, and lobules of squamous cells with prominent palisading interspersed with stellate reticulum, clusters of anucleate squamous cells, and myxoid microcysts. An immunohistochemical stain for β-catenin will demonstrate at least focal nuclear labeling (**B**). Papillary craniopharyngiomas (**C**) consist of fibrovascular cores lined by mature squamous epithelium and tumor-infiltrating neutrophils. Adamantinomatous and papillary craniopharyngiomas have distinct methylation profiles, as shown on this t-stochastic neighbor embedding (t-SNE) plot (**D**).

Adamantinomatous and papillary craniopharyngiomas both arise from Rathke’s cleft remnants and show squamous differentiation; thus, they were once considered histologic variants of the same tumor [9]. The 5th edition of the World Health Organization (WHO) Classification of Central Nervous System Tumors classifies them as distinct tumor types due to their distinct morphology and molecular underpinnings [9]. The epithelium of ACP is arranged in cords, lobules, and whorls with prominent palisading intermixed relatively paucicellular microcystic areas of stellate reticulum (Figure 2A). Degenerative features such as microcysts filled with myxoid material, anucleate remains of squamous cells (“wet keratin”), and calcifications are usually present (Figure 2A). Xanthogranulomatous reaction to ruptured cyst material, which is characterized by cholesterol clefts, hemosiderin, lymphohistiocytic infiltrates, and multinucleated giant cells, is a frequent finding in the adjacent tissue and sometimes composes the majority of small biopsy specimens [9]. In nearly all cases, these tumors are driven by *WNT* pathway activation via somatic *CTNNB1* mutations [9], which can be demonstrated by using immunohistochemistry to confirm nuclear and cytoplasmic labeling with β-catenin. Nuclear labeling with β-catenin is often focal (Figure 2B), and next-generation sequencing can be used to confirm these *CTNNB1* mutations.

Papillary craniopharyngiomas, which are driven by BRAF p. (V600E) mutations, are composed of broad fibrovascular cores lined by mature squamous epithelium with tumor-infiltrating neutrophils (Figure 2C) and patchy lymphohistiocytic infiltrates. The nuclear palisading, stellate reticulum, calcifications, and degenerative features seen in ACPs are absent in papillary craniopharyngiomas. Methylation profiling also demonstrates epigenetic differences between adamantinomatous and papillary craniopharyngiomas (Figure 2D). A subset of cases shows clusters of goblet cells within the squamous epithelium or even regions of ciliated epithelium, which can make them difficult to distinguish from Rathke Cleft cysts with extensive squamous metaplasia [9]. Next-generation sequencing or immunohistochemistry demonstrating a BRAF p. (V600E) mutation can be used to confirm the diagnosis.

CPs present significant diagnostic and management challenges across all age groups, from prenatal to adult cases. Early and accurate diagnosis, along with tailored surgical and multidisciplinary interventions, play a crucial role in improving patient outcomes. Despite advances in detection and treatment, issues such as recurrence, hypothalamic-pituitary dysfunction, and long-term quality-of-life concerns persist. Ongoing research and refined treatment strategies are essential to enhance the survival rates and quality of life for patients affected by this complex tumor. Continued efforts in early diagnosis, effective surgical techniques, and comprehensive follow-up care will be pivotal in addressing the multifaceted challenges posed by craniopharyngioma.

The radiographic appearance of CPs is dependent on their subtype. The adamantinomatous subtype typically presents in the pediatric and adult age groups, demonstrating a variable degree of calcifications within the tumor and classically multiple cyst walls [63]. They typically involve the pituitary stalk and extend inferiorly into the subdiaphragmatic space. On MRI, ACPs may display highly variable intensities and enhancement patterns given the likelihood of mixed cystic and solid components. The papillary variant, predominant in the adult age group, does not typically have calcifications on CT [53]. Although papillary subtypes may have a single small cystic component, they are typically solid, demonstrate avid enhancement, and extend into the third ventricle [53,63].

## 8. Treatment

### 8.1. Targeted Therapies for Craniopharyngiomas Based on Genetic Mutations

The identification of specific genetic mutations in CPs has paved the way for targeted therapeutic strategies that address the underlying molecular mechanisms driving tumor growth. Unlike conventional treatments that frequently lead to collateral damage to nearby healthy tissues, targeted therapies are designed to specifically inhibit the molecular pathways responsible for tumor growth. This precision enables a reduction in treatment-related complications and helps to preserve essential neurological and endocrine functions [64]. For ACPs, which are characterized by CTNNB1 mutations, research into therapies that target the Wnt/β-catenin signaling pathway is ongoing. Although no targeted treatments are yet approved specifically for this subtype, inhibitors of the Wnt pathway, such as those targeting β-catenin or its downstream effectors, are being explored in preclinical models and early-phase clinical trials [65]. These therapies aim to disrupt the aberrant signaling that promotes tumor proliferation and survival.

In the case of PCPs, the presence of the BRAF V600E mutation has led to the development of approved targeted treatments using BRAF inhibitors. Drugs such as vemurafenib and dabrafenib, which are already approved for other BRAF-mutant cancers, have shown promise in clinical trials for PCPs [11,12,13,64,65,66]. These inhibitors work by blocking the mutated BRAF protein, thereby inhibiting the MAPK/ERK signaling pathway and reducing tumor growth. The application of these targeted therapies not only offers a more personalized approach to treatment but also highlights the potential for significant therapeutic advancements in managing CPs. However, some studies have found an activation of the MAPK pathway following BRAF inhibitor monotherapy, which has been linked to developing resistance to treatment within 6–7 months [67,68,69]. Combination therapy has been shown to prevent or delay this acquired resistance when BRAF inhibition is paired with a mitogen-activated extracellular signal regulated kinase (MEK) inhibitor [69,70]. Targeted agents may also incur adverse events, including rash, fatigue, diarrhea, peripheral edema, arthralgias, pyrexia, and anorexia [69,71]. Continued research and clinical trials are essential to optimize these treatments and expand their efficacy, ultimately improving patient outcomes.

### 8.2. Surgery

Currently, surgical resection is the mainstay treatment for CPs [72,73]. Meta-analyses have demonstrated increased recurrences rates with STR when compared to GTR [72,73]. Controversially, GTR and STS + Radiation Therapy (RT) patients have been reported to have comparable long-term survival rates and postoperative complications [74]. Surgical corridors may be created via a transcranial approach, endoscopic endonasal transsphenoidal approach (EEA), and, in certain cases, a combined approach for exceptionally large craniopharyngiomas [72]. Transcranial approaches (TCAs) may include pterional, supraorbital, interhemispheric, subfrontal/bifrontal, modified orbitozygomatic, and transpetrosal corridors that are individualized based on the specific goals of surgery. EEA and TCA, at very experienced centers, can offer even patients with giant craniopharyngiomas (greater than 4 cm in maximal dimension) safe and effective treatments that decrease tumor burden [75]. Advancements in intraoperative technology and equipment, including intraoperative MRI, have led to improvements in surgical resection rates [76].

**Figure 3:**
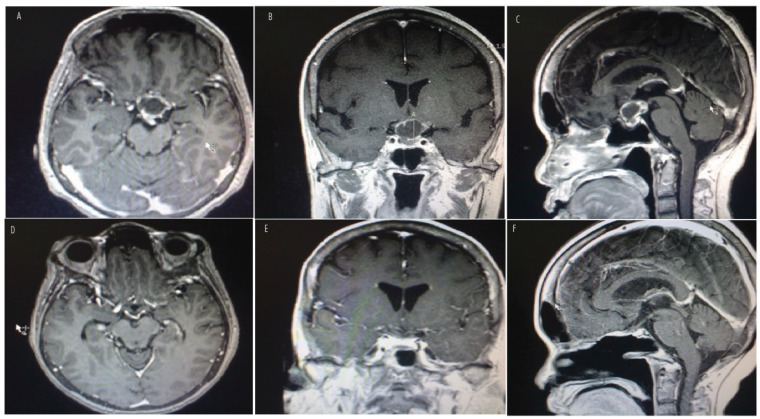
Preoperative axial, coronal, and sagittal MR images with contrast (**top row**, **A**–**C**) of a 12-year-old boy presenting with peripheral vision loss and diabetes insipidus. Physical examination revealed a bitemporal hemianopsia. Imaging demonstrated a predominantly multicystic and solid suprasellar mass extending inferiorly into the subdiaphragmatic space, causing a chiasmatic mass effect that was partially calcified on CT imaging. The patient underwent an endoscopic endonasal approach with gross total resection with vascularized nasoseptal flap for skull base dural reconstruction. Postoperative MR imaging (**bottom row**, **D**–**F**) revealed chiasmatic decompression and no residual tumor. His vision improved and final pathology revealed adamantinomatous craniopharyngioma.

**Figure 4:**
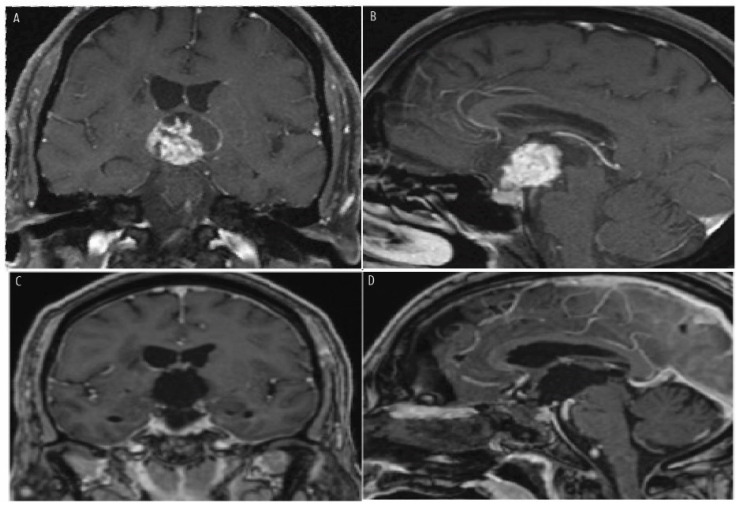
Preoperative coronal, and sagittal MR images with contrast (**top row**, **A**,**B**) of a 62-year-old woman presenting with peripheral vision loss. Physical examination revealed a bitemporal hemianopsia. Imaging demonstrated a cystic and predominantly solid suprasellar mass extending superiorly into the third ventricle, causing a chiasmatic mass effect that did not have calcifications on CT imaging. The patient underwent an endoscopic endonasal approach with gross total resection with vascularized nasoseptal flap for skull base dural reconstruction and preservation of the normal pituitary gland. Postoperative MR imaging (**bottom row**, **C**,**D**) revealed chiasmatic decompression, preservation of the pituitary gland, and no residual tumor. Her vision improved and final pathology revealed papillary craniopharyngioma.

Figure 3 and Figure 4 show preoperative MR images with contrast of a 12-year-old boy (Figure 3A–C) and a 62-year-old woman (Figure 4A,B) presenting with peripheral vision loss and bitemporal hemianopsia. Both patients had cystic and solid suprasellar masses causing chiasmatic mass effect, with partial calcifications noted in the boy’s CT imaging. Both underwent endoscopic endonasal resection with a vascularized nasoseptal flap for skull base dural reconstruction. Postoperative MR images (Figure 3D–F for the boy; Figure 4C,D for the woman) demonstrate successful chiasmatic decompression, no residual tumor, and improved vision. Final pathology for the boy revealed adamantinomatous craniopharyngioma, while the woman’s pathology revealed papillary craniopharyngioma.

Although a meta-analysis by Elliott et al. in 2011 found that the endoscopic endonasal approach attained a higher GTR rate than the TCA, this difference was not shown to be statistically significant in a larger meta-analysis by Li et al. in 2024 for adult (OR  =  1.84, 95% CI 0.41–8.16, *p*  =  0.424) or mixed (OR  =  2.45, 95% CI 0.87–6.87, *p*  =  0.066) groups [74,77]. Similarly, the risk of recurrence was not statistically significant for EEA when compared to TCA (OR  =  0.41, 95%CI: 0.03–5.24, *p*  =  0493). This lack of significant difference between TCA and EEA has also been proposed to have an experience component, as the EEA is relatively more novel to TCAs, and reports have found EEA GTR rates are significantly higher for more senior/experienced surgeons than for surgeons with a limited clinical practice (71 vs. 47% *p* < 0.05) [78].

The EEA, when compared to TCA, has been found to significantly improve postoperative surgical outcomes, including decreased hypopituitarism (14.3% vs. 37.2%, OR  =  0.50, 95%CI: 0.28–0.88, *p*  =  0.016), postoperative hydrocephalus (2.0% vs. 24.3%, OR  =  0.06, 95%CI: 0.05–0.07, *p*  <  0.001), stroke (7.4% vs. 11.6%, OR  =  0.58, 95%CI: 0.51–0.66, *p*  <  0.001), infection (1.2% vs. 3.7%, OR  =  0.32, 95%CI: 0.24–0.42, *p*  <  0.001), visual deficit (4.6% vs. 13.6%, OR  =  0.30, 95%CI: 0.26–0.35, *p*  <  0.001), and mortality (7.4% vs. 11.6%, 0.58, 95%CI: 0.51–0.66, *p*  <  0.001) [74]. A large systematic review of 3470 patients and 88 studies comparing the approaches further demonstrated that EEA had statistically significantly higher rates of gross total resection (67% vs. 48%), better visual outcomes (56% vs. 33%), and higher rates of cerebrospinal fluid leakage (18% vs. 2.6%), while TCA was associated with higher rates of hypopituitarism and seizures [79].

In patients who underwent EEA, anterior pituitary deficits were more common in first operations when compared to reoperation (51% and 23%, respectively; *p* = 0.08), whereas new DI was more common in reoperated patients compared with first-time operations (80% vs. 47%, respectively; *p* = 0.08) [80]. Although the risk of CSF leak is higher for EEA than TCA (3.0% vs. 1.1%, OR  =  2.80, 95%CI: 2.11–3.72, *p*  <  0.001), this risk has also been shown to be acceptable with a pooled incidence of 3.0%, which may be further mitigated with use of a perioperative lumbar drain (OR  =  3.0, 95%CI 1.2–7.6, *p*  =  0.017) [74,81]. One multi-institutional study noted that EEA provided safety and efficacy with stable-to-improved endocrinologic outcomes for residual and recurrent craniopharyngioma resection following subtotal resection through initial TCA [82].

The overall care for craniopharyngiomas must be individualized to the specific patient and goals of treatment. In general, our group believes in maximal safe surgical resection, favoring the EEA for the vast majority of craniopharyngiomas; in rare instances with substantial lateral extension, a TCA may be more suitable. The endonasal approach provides excellent visualization of the superior hypophyseal arteries, infundibulum, hypothalamus, and undersurface of the optic apparatus and allows for the resection of the tumor with minimal to no retraction on normal brain tissue. Sharp dissection is paramount, and reconstruction methods must be meticulous. Any residual tumor in surgically inaccessible or potentially unforgiving locations, such as the hypothalamus, can be managed with surveillance imaging and additional nonsurgical treatments, such as radiation therapy and approved BRAF inhibitors for the papillary variant. Significant preoperative discussions with the patient and treating surgeon must include the patient’s perspective on the acceptance of potentially having panhypopituitarism if surgical cure is likely.

Pediatric patients pose additional surgical challenges that need to be recognized. Children less than 10 years of age may have an incomplete pneumatization of the paranasal sinuses and may require significant additional skull base drilling to achieve an adequate surgical corridor through the EEA [83,84]. In these cases, CT angiography scans are crucial to limit vascular injury [83]. Additionally, the complications of hypopituitarism in this population are significant. CT and MR imaging must be carefully reviewed to identify the location of the craniopharyngioma and its relationship to the normal pituitary gland and optic chiasm. Craniopharyngiomas with large cystic portions are often seen in this population. One general strategy in very young patients may be to effectively treat presenting symptoms through cyst fenestration, aspiration, and intracavitary treatments through open approaches and subtotal resections. These may delay full resection through EEA until after they reach the late-teenage years to have maximum paranasal sinus pneumatization, in order to limit the consequences of prepubertal hypopituitarism. In general, we try to avoid radiation therapy in the pediatric population given the possibilities of hypopituitarism, loss of tissue planes with future surgeries, radiation-induced neoplasms, and neurovascular injury. We have summarized the challenges of the surgical approaches in the pediatric population in Table 3.

### 8.3. Intracavitary Adjuncts

Intracavitary beta–gamma radiation remains the most established and well-described intracystic treatment option for craniopharyngiomas, with volume reduction seen in up to 80–100% cases [72,85,86]. Isotopes of phosphorus, aurum, rhenium, and yttrium may be introduced via an Ommaya reservoir device. Isotopic dose may range from 200–400 Gy. However, potential complications may occur, including the induction of secondary tumors, off-target radiotoxicity to surrounding vital structures, and delayed onset vasculopathies, including moyamoya disease [72,86,87,88].

Similarly, bleomycin, a polypeptide DNA-/RNA-inhibiting antibiotic extracted from Streptomyces verticillus, and IFN-alpha, which increases cytokine secretion and T-cell activation, may be administered via an Ommaya reservoir and be used for volume control with CPs [7,18,19,20,22]. These medications provide additional options for local tumor control in populations sensitive to radiation therapy [72,88,89,90,91].

### 8.4. Radiation Therapy

Although GTR may be curative, STR followed by RT (photon or proton beam) may be a safe alternative, particularly when CPs exhibit invasion into surrounding structures, including the hypothalamus [92]. Through this preservation of the HPA axis, the frequency of long-term complications, including severe obesity and diabetes insipidus, may be decreased [93,94]. Some studies have demonstrated a similar risk of developing recurrence for GTR vs. STR  +  RT (odds ratio: 0.63, 95%CI: 0.33–1.24, *p*  =  0.18) [95]. Theoretically, proton beam therapy deposits energy at a more precise defined point and limits radiation around the tumor, thereby minimizing harmful radiation effects to surrounding structures; this specificity is particularly utilized in pediatric populations. Excellent 5-year local tumor control rates of 91.1% have been reported by Jimenez et al. for these pediatric craniopharyngioma patients undergoing postoperative proton beam therapy [72,96]. When compared to photon-based RT, radiotherapy-associated toxicity may potentially be decreased with proton beam therapy [97,98]. Additionally, certain studies have found improved functional outcomes in children with craniopharyngiomas treated with proton beam therapy, as academic achievement scores (math and reading) did not change significantly after proton beam therapy, but a significant decline was noted in patients treated with photon-based RT [98,99]. Photon-based RT methods with Gammaknife and Cyberknife have been reported to have a 62–90% five-year local tumor control and 85% three-year local tumor control, respectively [100,101]. In Table 4, we provide a comprehensive summary of the descriptions, advantages, and limitations of each treatment modality.

### 8.5. Multidisciplinary Approach

A collaborative multidisciplinary center with primary care physicians/pediatricians, neuroradiologists, neurosurgeons, neuropathologists, otolaryngologists, neuro-oncologists, endocrinologists, ophthalmologists, and neurologically focused radiation oncologists is paramount to adequately treat CPs [2,3]. Their rarity and proximity to key sellar/parasellar structures make expertise in operative and nonoperative management essential [1]. Despite their benign histology, the surgeon must understand the anatomy of the tumor and key nearby structures, including the internal carotid arteries and its branches, structures of the hypothalamic–pituitary axis, and optic and oculomotor nerves prior to resecting these lesions [2,3]. Similarly, the surgeon must understand when the benefit of achieving GTR may not outweigh the risk of iatrogenic damage when resecting more invasive variants.

Additionally, the expertise of endocrinologists is frequently necessary to detect and treat new postoperative endocrinologic disturbances, including diabetes insipidus (DI), hypopituitarism, and hypothalamic obesity often associated with age at diagnosis, tumor recurrence or progression, and the ACP histological subtype [4,5]. A study investigating the postoperative DI incidence in pediatric and adult populations found that 80% of pediatric patients and 63% of adults developed DI after craniopharyngioma resection [102]. In addition, a meta-analysis and systematic review of the literature reported that post-operative DI was highest in patients undergoing craniopharyngioma resection compared to other pituitary region tumors [103]. Acute and chronic management is essential to address potentially life-threatening consequences, including electrolyte imbalances, hypovolemia, renal damage, and neurological abnormalities [104]. Another endocrinopathy that may arise following craniopharyngioma resection is hypothalamic obesity, due to tumor hypothalamic invasion or damage during resection. A study found that patients who underwent hypothalamic sparing surgery had a lower risk of obesity compared to patients with extensive resection surgery [93].

Additional hypopituitarism following craniopharyngioma surgical resection can occur. Patients may experience other hormonal deficiencies, including growth hormone (47–93%), adrenocorticotropic hormone (43–92%), luteinizing hormone/follicle-stimulating hormone (61–91%), and thyroid-stimulating hormone (50–86%). Some studies report three or more hormonal deficiencies in 75–100% of patients [105]. The endocrine management of hypopituitarism following craniopharyngioma resection is crucial in ensuring that patients receive adequate hormonal replacement therapy and comprehensive long-term care.

In addition, the potential for postoperative CSF leaks must be closely monitored. A retrospective study reported that the post-operative risk for experiencing a CSF leak following craniopharyngioma resection was 4.7% [106]. Endoscopic transsphenoidal approaches were associated with an increased risk (18.4%) compared to microscopic transsphenoidal (9%) and transcranial approaches (2.6%) [79]. Bacterial or aseptic meningitis may follow a CSF leak [107]; therefore, it is very important to employ a multidisciplinary approach in correctly identifying and appropriately treating these complications.

**Figure 5:**
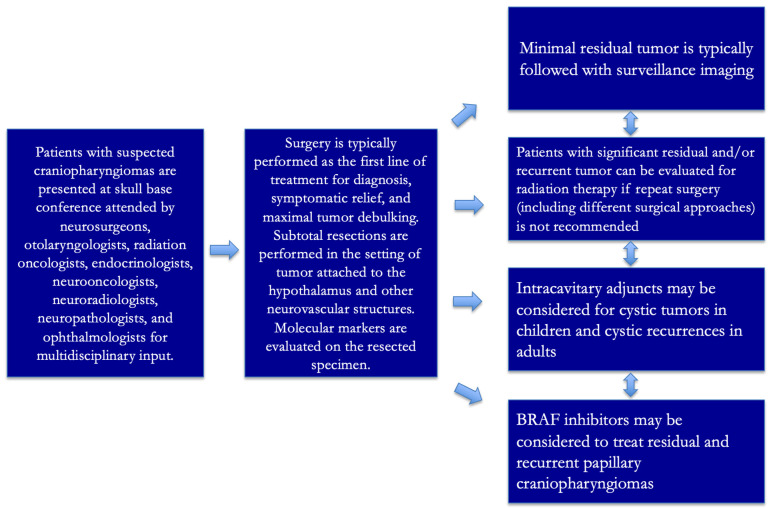
General multidisciplinary treatment strategy at our institution.

Craniopharyngioma treatment is very individualized given the variety of types, solid and cystic components, locations, presenting symptoms and signs, patient wishes, patient demographics/comorbidities, and available multidisciplinary treatments. Periodic postoperative surveillance imaging is necessary. With residual and recurrent CPs, consideration for medical therapy, radiation therapy (photon or proton beam), and even re-resection through similar or different approaches must be considered [8]. We have summarized our group’s general clinical treatment strategy in Figure 5.

## 9. Prognosis and Long-Term Outcomes

The complex nature of CP, combined with its treatment requirements, often results in lasting consequences that affect various aspects of health and well-being. In this context, a review of CP survivors from 2000 to 2019 reveals critical insights into the long-term effects of this condition [108]. Initial ophthalmological findings showed that 70% of patients presented with visual impairment at diagnosis, with 32% demonstrating improvement following surgical intervention. Persisting visual impairment correlated with decreased social functioning and autonomy in long-term follow-up assessments, underscoring the necessity for early detection and tailored aftercare strategies to enhance the quality-of-life outcomes in CP management [108].

Additionally, the recurrent nature of pediatric CP presents significant challenges. Identifying key prognostic factors is crucial for developing effective management strategies and improving patient prognoses. A recent review on pediatric CP recurrence has shed light on several critical factors influencing outcomes [109]. Incomplete surgical resection by an experienced neurosurgical team and the omission of post-subtotal excision radiotherapy significantly increased recurrence rates. Additional risk factors included younger age at diagnosis (<5 years), larger tumor size, cystic morphology, challenging tumor location, and histological features such as adherence to adjacent structures. However, conflicting data regarding other potential risk factors highlights the ongoing need for refined management strategies [109]. These insights underscore the necessity of targeted approaches to optimize outcomes for children with CPs. A recent investigation into pediatric patients with CPs provided valuable insight into the disease’s trajectory and treatment efficacy [110]. With a median follow-up of 6.60 years, common initial symptoms included raised intracranial pressure (58.3%), visual deficits (50.0%), and preoperative endocrine abnormalities (16.7%). Treatment outcomes indicated that 41.7% underwent gross total resection and 58.3% underwent subtotal resection. Overall survival was observed in 75.0% of patients, with 58.3% experiencing recurrence at a median time of 5.87 months, and a median progression-free survival of 4.16 years [110]. This study highlights the significant challenges in managing pediatric CPs, characterized by frequent recurrences and enduring neuroendocrine complications that impact the long-term quality of life. These findings underscore the imperative for advancing therapeutic strategies to improve outcomes and mitigate the burden of disease for affected children.

Understanding the effects of surgical treatment on hypothalamic–pituitary dysfunction (HPD) in CP patients is crucial for optimizing patient outcomes and managing long-term complications. A recent retrospective study involving 742 CP patients provides valuable insights into these effects over a median follow-up of 15 months [111]. The study revealed notable differences between PCP and ACP in terms of preoperative and postoperative dysfunction. PCP patients exhibited higher rates of diabetes insipidus (DI) and hyperprolactinemia preoperatively, while ACP patients more frequently presented with adrenocorticotropic hypofunction. ACP tumors predominantly originated in the sellar region, contrasted with the suprasellar location of PCP tumors. Post-surgery, both groups showed increased rates of adenohypophyseal hypofunction, DI, and hypothalamic obesity compared to baseline, with ACP patients experiencing more pronounced effects. Factors such as older age at diagnosis, tumor recurrence or progression, and the ACP histological subtype were identified as significant contributors to worsened HPD after surgery. These findings underscore the importance of targeted surgical strategies and follow-up care to mitigate the impact of HPD in craniopharyngioma patients [111].

Adult CPs present a complex challenge in clinical management, necessitating a thorough understanding of treatment outcomes to improve patient survival. Results from a study utilizing the 2004–2018 National Cancer Database offer critical insights into the management of these tumors in adults. Among 666 patients with a mean age of 51 years, multivariable analysis highlighted factors independently associated with poorer overall survival (OS), including older age, uninsured status, Medicaid or Medicare coverage, higher Charlson–Deyo Comorbidity Index, and larger tumor size (>40 mm) [112]. Importantly, gross total resection (GTR) and subtotal resection (STR) with adjuvant radiotherapy were independently linked to improved OS. Although GTR with radiotherapy showed a trend towards enhanced OS, STR alone and radiotherapy alone did not significantly differ from no treatment in survival outcomes. Kaplan–Meier survival analyses corroborated these findings, demonstrating superior survival with GTR, GTR + radiation therapy, and STR + radiation therapy regimens [112]

## 10. Conclusions and Prospects

Craniopharyngiomas are very locally aggressive tumors in critical areas of the brain. While significant improvements in diagnostic and therapeutic modalities have been made since Dr. Cushing initially coined the term, craniopharyngiomas require multidisciplinary treatments that still inherently carry risk to the patient. Experiences and analyses into surgical approaches and new intraoperative technology continue to advance resection capabilities; however, with adherent tumor to critical parts of the brain often mandating subtotal resections and tumor recurrences, non-surgical therapies, including radiation therapy, play a significant role. Relatively recent advancements in identifying genetic mutations have led to burgeoning targeted therapies. The future likely holds promising advances for early and accurate diagnosis, improved surgical and radiation techniques with less morbidity, and new molecular targeted therapies that will continue to redefine what is considered the standard of care for these rare tumors.

## Figures and Tables

**Table 1:** General differences in types of craniopharyngioma.

	Adamantinomatous Variant	Papillary Variant
Age group	Children and adults	Adults
Location	Pituitary stalk, subdiaphragmatic	Pituitary stalk, third ventricle
Texture	Calcified Multicystic components	Not calcified Predominantly solid
Hypothalamic and pituitary gland invasiveness	More with tumor noted to usually be very adherent to neurovascular structures	Less with tumor noted to usually be less adherent to neurovascular structures
Mutation	*CTNNB1* (chromosome 3p21) with upregulation of Wnt-β-catenin signaling pathway genes (including EGFR- and SHH-)	*BRAF V600E* (chromosome 7q34) with upregulation of MAPK/ERK signaling pathway genes

**Table 2:** Most common symptoms and presenting signs [56].

Symptom/Sign	Frequency
Visual impairment (temporal hemianopsia most common from chiasmatic compression)	62–84%
Endocrine dysfunction (from involvement/compression of the pituitary stalk)	40–87%
Growth Hormone deficiency (causing weight gain, obesity, fatigue)	85%
Gonadotroph deficiency (causing amenorrhea, loss of libido, erectile dysfunction)	40%
ACTH deficiency (causing dizziness, hypotension, weight loss)	25%
TSH deficiency (causing weight gain, constipation, cold intolerance)	25%
Vasopressin deficiency (causing diabetes insipidus)	20%
Headaches	50%

**Table 3:** Challenges of surgical approaches in the pediatric population.

Approach	Challenges/Limitations
TCA	Need for brain retraction Less visualization of small perforator vessels providing blood supply to the undersurface of the optic chiasm and pituitary stalk Less visualization of the hypothalamus Lower rates of gross total resection Higher rates of seizures
EEA	Incomplete pneumatization of the paranasal sinuses Smaller working corridor, particularly with an underdeveloped sella or prefixed chiasm Increased risk of hypopituitarism with anteriorly and inferiorly displaced normal glands Higher rate of postoperative cerebrospinal fluid leak and need for multilayered skull base reconstruction

**Table 4:** Advantages and disadvantages of treatment modalities for craniopharyngioma.

Treatment Modality	Description	Advantages	Disadvantages/Limitations
Surgery	Subtotal or complete resection via the TCA or EEA	-tumor volume reduction and diagnosis -decreased RT dose post-resection -multiple corridors (TCA vs. EEA) with varying risk profiles -standard of care for adult and pediatric populations -potentially curative after GTR	-invasive -recurrence and risk for reoperation after STR -risk of endocrinologic disruption requiring hormone replacement -patient must meet surgical criteria and hold blood thinners perioperatively -added complexity in children 0–10 years old (incomplete paranasal sinus pneumatization)
Radiation Therapy	Stereotactic radiosurgery or proton beam RT (preferred in children)	-noninvasive -targets postop residual invasive CP -precise targeting with proton beam used in children -excellent local tumor control (up to 91.1%)	-limited use in pediatrics (radiosensitive) -radiotoxicity -may induce secondary tumors -delayed onset vasculopathies
Targeted Therapies	Genetic targets (CTNNB1 and BRAF V600E mutations) identified to interrupt molecular mechanisms of CP growth	-noninvasive -potentially decreased damage to non-CP tissue and preserved endocrinologic functioning -options for combination therapy	-requires crossing blood-brain barrier -potential acquired resistance -clinical trials ongoing, not currently approved for routine use in ACP treatment -adverse medication event
Intracavitary Treatments	Beta–gamma radiation and/or bleomycin	-additional volume reduction, particularly for cystic portions, after surgical resection	-may induce secondary tumors -radiotoxicity -delayed onset vasculopathies -limited radiation use in children (radiosensitive)
